# Survival of viral haemorrhagic septicaemia virus and infectious haematopoietic necrosis virus in the environment and dried on stainless steel

**DOI:** 10.1111/tbed.13888

**Published:** 2020-11-07

**Authors:** Claire L. Joiner, Birgit C. Oidtmann, Georgina S. E. Rimmer, Nicola J. McPherson, Peter F. Dixon, Richard K. Paley

**Affiliations:** ^1^ Centre for Environment Fisheries and Aquaculture Science Weymouth UK; ^2^ Department for Environment, Food and Rural Affairs London UK

**Keywords:** adsorption, environment, IHNV, stainless steel, survival, VHSV

## Abstract

Viral haemorrhagic septicaemia virus (VHSV) and infectious haematopoietic necrosis virus (IHNV) are important viral pathogens posing a serious threat to salmonid fish. Survival of two isolates of IHNV and one of VHSV was assessed at temperatures ranging from 4 to 25°C: (a) after drying on stainless steel, (b) in cell culture medium, (c) in filtered river water, (d) in unfiltered river water, and (e) survival, adsorption and desorption in river sediment and five typical soil types. The viruses survived 1 hr to > 84 days depending on the conditions. Survival was inversely related to temperature and organic and inorganic content. Both viruses remained infectious after being dried on stainless steel for several weeks highlighting the risk of mechanical transmission and persistence in a dry environment. Both adsorbed to the soils from the river water inoculum, with titres between 5.56x10^4^ and 2.58x10^8^ TCID_50_/ml after 1 hr. Clay soils adsorbed the least virus but had the greatest decrease in the river water inoculum (undetectable in ≤ 1 hr), and there was no desorption. Virus desorbed from the other soils into the surrounding water at different rates dependant on soil type (longest desorption was from chalk loam and sandy soil—detected at 28 days). When desorption was no longer detectable, virus persisted, adsorbed to the soil and remained infectious (the longest adsorption was detected in clay loam for ≥ 49 days, but all the viruses adsorbed to soils were likely to have survived longer than that detected, based on their rate of decay). The long survival of the viruses, particularly at cooler temperatures, highlights the risk of survival in the environment and waterborne spread. The data presented here are highly relevant for assessing risk of pathogen introduction via fomites (stainless steel) and for deciding on best control measures in the context of disease outbreaks.

## 1 INTRODUCTION

The rhabdoviruses viral haemorrhagic septicaemia virus (VHSV) and infectious haematopoietic necrosis virus (IHNV) are the cause of serious diseases in finfish worldwide due to their pathogenicity, disease course, the hosts’ importance in aquaculture and high mortality rates. Both pathogens are notifiable to the World Organization for Animal Health (OIE, 2019), and specific legislation to control these diseases is in place in several regions of the world, where salmonid fish are reared for aquaculture (e.g. under European Union Council Directive 2006/88/EC (Anon, 2006) and 91/67/EEC (Anon, 1991). The United Kingdom is currently free from both viruses (Anon, 2009); therefore, it is important to gather information to inform control measures in the event of disease introduction.

VHSV is currently present in Europe, oceans of the northern hemisphere, Asia and North America. Disease generally occurs at water temperatures between 4°C and 14°C. At temperatures above this, up to 18°C, the disease generally occurs over a shorter period with less mortality (OIE, 2019). VHSV has a wide number of hosts; it is currently known to have been isolated from approximately 80 fish species in the Northern Hemisphere and has caused significant losses in farmed rainbow trout, *Oncorhynchus mykiss,* in Europe and more recently also in a wide range of freshwater species in North America (OIE, 2019).

Infectious haematopoietic necrosis (IHN) was originally identified as a disease mainly of salmon and trout in enzootic areas of western North America, caused by the virus, IHNV. IHNV has since spread and is present in Europe, North America, Russia and several South East Asian countries. Experimental trials have demonstrated IHNV can produce mortality from 3°C to 18°C (Bootland & Leong, 1999); however, clinical disease typically occurs between 8°C and 15°C in farmed fish (Dixon et al., 2016; OIE, 2019). IHNV has been reported to naturally infect salmonids of the genus *Oncorhynchus* and Atlantic salmon (*Salmo salar*). Other salmonid species and a range of non‐salmonid species have occasionally been found to be infected in the wild or shown to be somewhat susceptible by experimental infection (Dixon et al., 2016; OIE, 2019).

Rainbow trout and salmon are the dominant freshwater salmonid species farmed in Europe and North America. There was approximately 850,000 tonnes of global aquaculture production of rainbow trout worth nearly 4,000 M USD and 2.6 M tonnes of salmon worth 18.5 M USD 1,000 in 2018 (retrieved from FAO FishStatJ—http://fao.org/fishery/statistics/software/fishstatj/en accessed 18 August 2020). With both fish species being highly susceptible to both viruses and with no treatment available, VHSV and IHNV outbreaks could have major economic impact on recreational angling and aquaculture businesses rearing salmon and rainbow trout. The European Reference Laboratory for Finfish Diseases collates data on disease outbreaks in Europe, and conservative estimates indicate that 75 and 46 salmonid farms were classed as ‘known to be infected’ for VHSV and IHNV, respectively, in 2017 (retrieved from http://www.eurl‐fish.eu/Activities/survey_and_diagnosis 16 March 2020).

In view of the fact that VHS and IHN are diseases having a major impact on salmonid aquaculture, additional environmental persistence data would benefit models of disease transmission. In this study, additional data were gained through specific experimentation to investigate the survival of isolates of VHSV and IHNV under different environmental conditions that may be expected in or around a typical salmonid farm or processing facilities: (a) after drying on stainless steel, (b) when mixed with cell culture maintenance medium (MM) (positive control), (c) in filtered river water (FRW), (d) in unfiltered river water (URW) and (e) survival, adsorption and desorption in river sediment (naturally occurring material that is broken down by processes of weathering and erosion) and five typical soil types. All mixtures were incubated at a range of temperatures from 4 to 25°C, a range to include both suboptimal and optimal temperatures for survival and infection. Because of the known effects of possible contamination, we also monitored the presence of fungi and bacteria visually during each experiment.

Phylogenetic analysis of VHSV has revealed four genogroups, I to IV, which have been divided into further subgroups (Ia‐e and IVa‐c; Einer‐Jensen et al., 2004; Snow et al., 2004). Subgroup Ia is most frequently associated with outbreaks of the disease in cultured rainbow trout (OIE, 2019). As such, the survival of the UK VHSV isolate J167 (from rainbow trout, 2006; Stone et al., 2008) of the genogroup Ia was studied. IHNV is divided into five genogroups, U, L, E, J and M, depending on the host species from which it is commonly isolated in various geographical areas (Cieslak et al., 2017; He et al., 2013; OIE, 2019). The M genogroup is based on the original North American geographic distribution and has evolved host specificity to rainbow trout (Kurath et al., 2003; Troyer & Kurath 2003). The E genogroup is a lineage of the M genogroup and has been found in Europe and Iran (Asl et al., 2007; Enzmann et al., 2010). The survival of the USA IHNV isolate HV‐90 (from rainbow trout 1991; LaPatra et al., 1991) and French IHNV isolate 32/87 (from rainbow trout, 1987; de Kinkelin et al., 1987) of the genogrouop M and E, respectively, were studied.

## 2 MATERIALS AND METHODS

### 2.1 Virus and cell culture

VHSV J167 (genogroup Ia) was propagated in *Epithelioma papulosum cyprini* (EPC) cells (Fijan et al., 1983) (ATCC^®^ CRL‐2872™), originating from common carp *Cyprinus carpio*, but shown to be contaminated with cells from fathead minnow (FHM) *Pimephales promelas* (Winton et al., 2010) at 15°C, in maintenance medium (MM) (Glasgow minimum essential medium supplemented with 10% foetal bovine serum (FBS), 1% L‐glutamine (200 mM solution), 0.16% sterile tris solution (120 g/L), 0.5% sterile 7.5% sodium bicarbonate and 1% penicillin‐streptomycin).

IHNV 32/87 (genogroup E) was propagated in EPC cells in MM (Glasgow minimum essential medium supplemented with 10% foetal bovine serum (FBS), 1% L‐glutamine (200 mM solution), 0.16% sterile tris solution (120 g/L), 0.5% sterile 7.5% sodium bicarbonate and 1% penicillin‐streptomycin). IHNV HV‐90, (genogroup M), was propagated in FHM cells (ATCC^®^ CCL‐42™), in MM (Minimum Essential Medium Eagle supplemented with 25 mM HEPES, 10% FBS, 1% L‐glutamine (200 mM solution), 1% MEM non‐essential amino acid solution (100x) and 1% penicillin‐streptomycin). (FHM cells were used for IHNV HV‐90 culture due to availability of cells during the study. Cell susceptibility and viral titres were sufficient to use this alternative cell line).

Virus stocks were prepared by propagating the viruses (previously stored at −70°C) and harvesting from monolayers showing full cytopathic effect (CPE). The harvests were clarified by low speed centrifugation (2000 x *g*, 5 min), and the supernatant containing the cultured virus was stored at 4°C and used within two weeks of harvest.

### 2.2 Virus quantification

Virus levels were quantified by titration in 96‐well micro‐titre plates with 6 wells per dilution. Twenty µl of sample was added to 180 µl of cell culture medium (to make a 1 in 10 dilution), and serial 10‐fold dilutions were made down the plate. Titrations were incubated in a humid chamber for 7 days at 15°C and end point 50% tissue culture infectious doses (TCID_50_/ml) were calculated (Kärber, 1931). The titres were calculated to account for the dilution of the virus before titration.

The stock viruses were quantified by titration at time 0 when initiating the experiments and read a week later.

### 2.3 Virus survival dried on stainless steel

**Figure 1:**
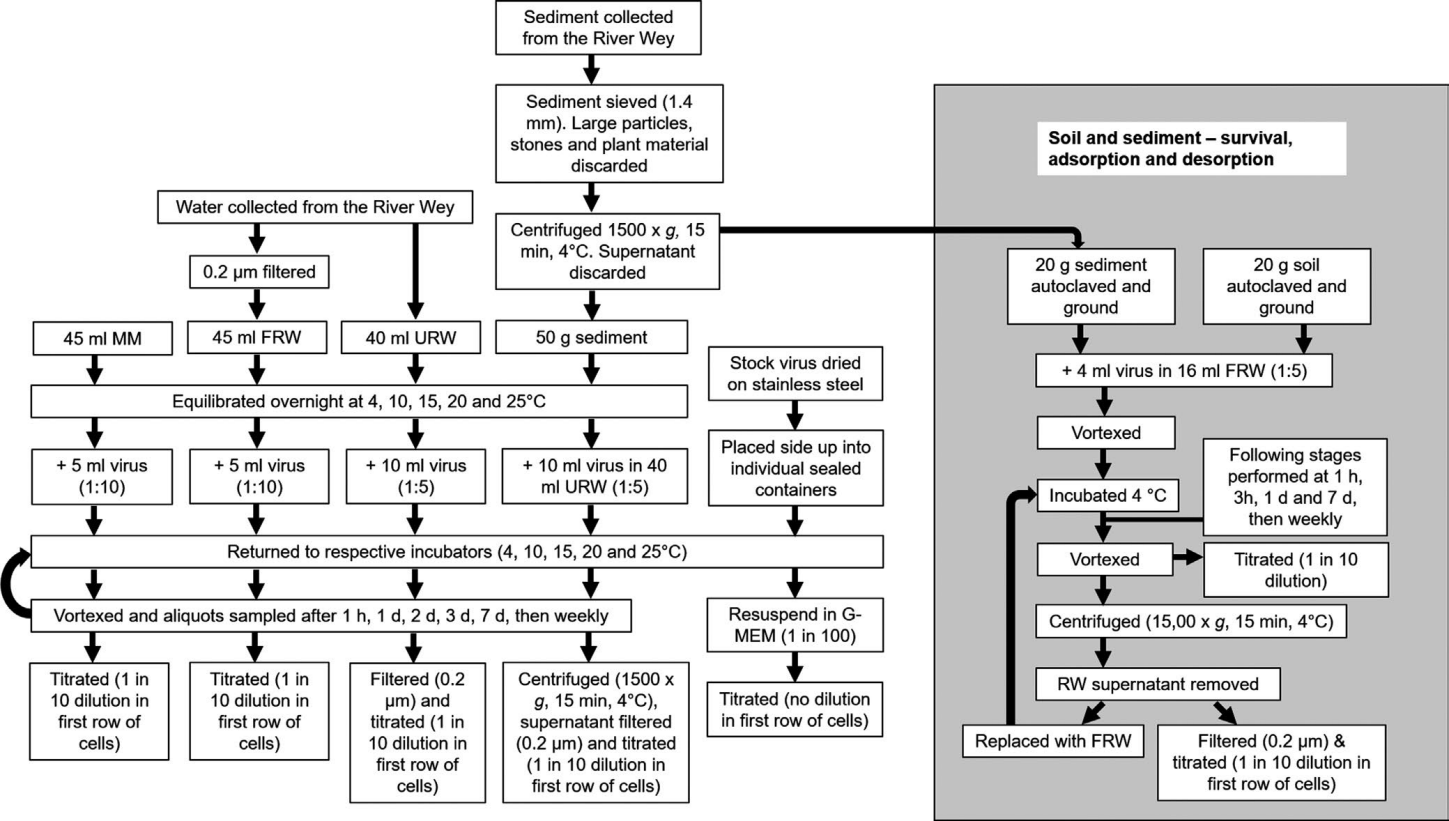
Flow diagram of the procedures used for assessing virus survival in cell culture maintenance medium (MM), filtered river water (FRW), unfiltered river water (URW), water separated from the unfiltered river water‐sediment mixture, resuspended virus after drying on stainless steel, and adsorption to soils and sediment mixtures

Fifteen µl of stock VHSV J167 (with an average titre [*n* = 2] of 1.48 × 10^9^ TCID_50_/ml), IHNV 32/87 (with an average titre [*n* = 2] of 1.48 × 10^8^ TCID_50_/ml) and IHNV HV‐90 (with an average titre [*n* = 2] of 3.38 × 10^6^ TCID_50_/ml) was added to autoclaved stainless steel discs, 1 cm in diameter (Figure 1). The virus was allowed to air dry in a class II biological safety cabinet, at room temperature (20°C ± 2°C), for 2 hr. Discs were placed in individual closed bottles with the dried virus side up and incubated at 4, 10, 15, 20 and 25°C. To determine the quantity of viable virus on the steel discs, at each sample point the dried virus on the discs was resuspended in 1.5 ml MM, vortexed for 10 seconds, placed on an orbital shaker at 500 rpm for 5 minutes then vortexed once more for 10 seconds. Medium was removed from the first row of cells of a 96‐well cell culture plate and replaced with 200 µl of the resuspended virus providing a 1 in 100 dilution on the first row. Serial dilutions were made from these as described above. Samples were taken day 0 (1 hr after spiking), days 1, 2, 3 and 7 then weekly intervals up to day 49 for VHSV and 56 for IHNV. Two discs were analysed at each sampling point for the first 3 weeks (and an average titre calculated) then reduced to one sample per time point. The limit of detection of the assay was 1.76x10^2^ TCID_50_/ml.

### 2.4 Virus survival in environmental matrices

Sterile MM, supplemented as described above, was used as a control matrix. River water and sediment samples were collected from the River Wey, Weymouth, Dorset, UK (SY 66,622 84,275) in October (when IHNV testing), September (when VHSV testing) and February (when testing both viruses for adsorption and desorption) . RW samples were unfiltered and filtered through a 0.2 µm filter with cellulose acetate membrane. Sediment was collected from the side of the riverbank where there was wet mud. Large stones and plant materials were removed; the sediment was sieved (1.4 mm mesh), and the sample centrifuged at 1,500 × *g* for 15 minutes at 4°C, excess water was discarded to provide a sample with minimal water present.

A single closed glass bottle containing either 45 ml of MM, 45 ml of FRW, 40 ml of URW or 50 g of river sediment was equilibrated overnight at each of five temperatures: 4, 10, 15, 20 and 25°C. The following day, virus suspensions were made in each matrix from stock virus stored at 4°C. Starting titres of stock viruses were determined as 3.78 × 10^8^ TCID_50_/ml for VHSV J167; 5.56 × 10^7^ TCID_50_/ml for IHNV 32/87 and 1.20 × 10^8^ TCID_50_/ml for IHNV HV‐90. A 1 in 10 dilution of virus was created by adding 5 ml stock virus to the MM and FRW, and 10 ml of the stock virus was added to the URW to create a 1 in 5 dilution. A 50 g sample of river sediment was mixed with 50 ml URW containing stock virus at a 1 in 5 dilution. Preparations were returned to their respective temperatures for incubation and sampling.

Samples were taken at day 0 (1 hr after spiking), days 1, 2, 3 and 7, then weekly intervals for up to 72, 65 and 84 days for IHNV 32/87, IHNV HV‐90 and VHSV J167, respectively, to assess virus survival over time. On two occasions (for IHNV 32/87 after day 56 and IHNV HV‐90 after day 49), the weekly sample interval was extended to 9 days. At each sampling, matrix suspensions were vortexed. For spiked MM and FRW, 20 µl aliquots were titrated directly onto cells as described in virus quantification above. 0.5 ml aliquots from the spiked URW were filtered (0.2 µm cellulose acetate membrane) immediately prior to titration to prevent any interfering microbial growth on the cells. Spiked sediment was mixed by manual agitation (inversion and swirling), 2 ml aliquots were centrifuged at 2060 × *g* for 15 minutes at 4°C and the supernatant filtered (0.2 µm cellulose acetate membrane) before titration. Mixtures were replaced back in their allocated incubators immediately after sampling. Once the titre of virus in a test matrix was no longer detectable for two consecutive weeks, titrations ceased for that matrix‐temperature combination. The limit of detection of the assay was 1.76 × 10^−1^ TCID_50_/ml.

### 2.5 Soil and sediment—survival, adsorption and desorption

Five dry typical soil types, clay soil, clay loam, chalk soil, chalk loam and sandy soil (Fisher Scientific™ UK), were autoclaved and ground in a pestle and mortar to remove large lumps and allow homogenous particle mixing (Figure 1). The river sediment was collected and processed as previously described. For each of the soils or sediment, 20 g was mixed with 4 ml (a) VHSV J167 (with starting titre 1.20 × 10^8^ TCID_50_/ml), (b) IHNV 32/87 (with starting titre 1.76 × 10^7^ TCID_50_/ml) and (c) IHNV HV‐90 (with starting titre 2.58 × 10^7^ TCID_50_/ml) and 16 ml FRW (equilibrated to 4°C). The mixtures were incubated at 4°C, and after 1 hr, 3 hr, 1 day, 7 days and then weekly, the mixtures were vortexed and the combined sediment‐FRW and soil‐FRW mixtures titrated directly to quantify the virus survival. The mixture was then centrifuged (1,500 × *g* for 15 minutes at 4°C) and the supernatant filtered (0.2 µm cellulose acetate membrane) and titrated. The supernatant was replaced with FRW (that had been sealed and stored at 4°C over the experimental period), after each titration to observe any desorption of virus from the soil into each wash. The same FRW was run alongside the experiment, 4 ml each virus was added to 16 ml FRW (1 in 5 of each virus) and maintained under the same conditions with the same sample points.

Once the titre of virus in a test matrix was no longer detectable for at least two consecutive weeks, titrations ceased. The limit of detection of the assay was 1.76x10^‐2^ TCID_50_/ml for the removed liquid but visibility was restricted for the soil mixture meaning the limit of detection was between 1.76 × 10^−3^ TCID_50_/ml and 2.58 × 10^−5^ TCID_50_/ml.

Initial adsorption, within the first hour, was calculated by removing the titre of the virus in the FRW inoculum from the titre in the FRW‐soil/sediment mixture at 1 hr. Survival of the adsorbed virus in the soils/sediment was also measured by ‘the titre of the virus in the combined FRW‐soil/sediment mixture’ minus ‘the titre of the desorbed virus’. This was log_10_ converted to calculate the log_10_ drop from that adsorbed in the first hour.

## 3 RESULTS

**Figure 2:**
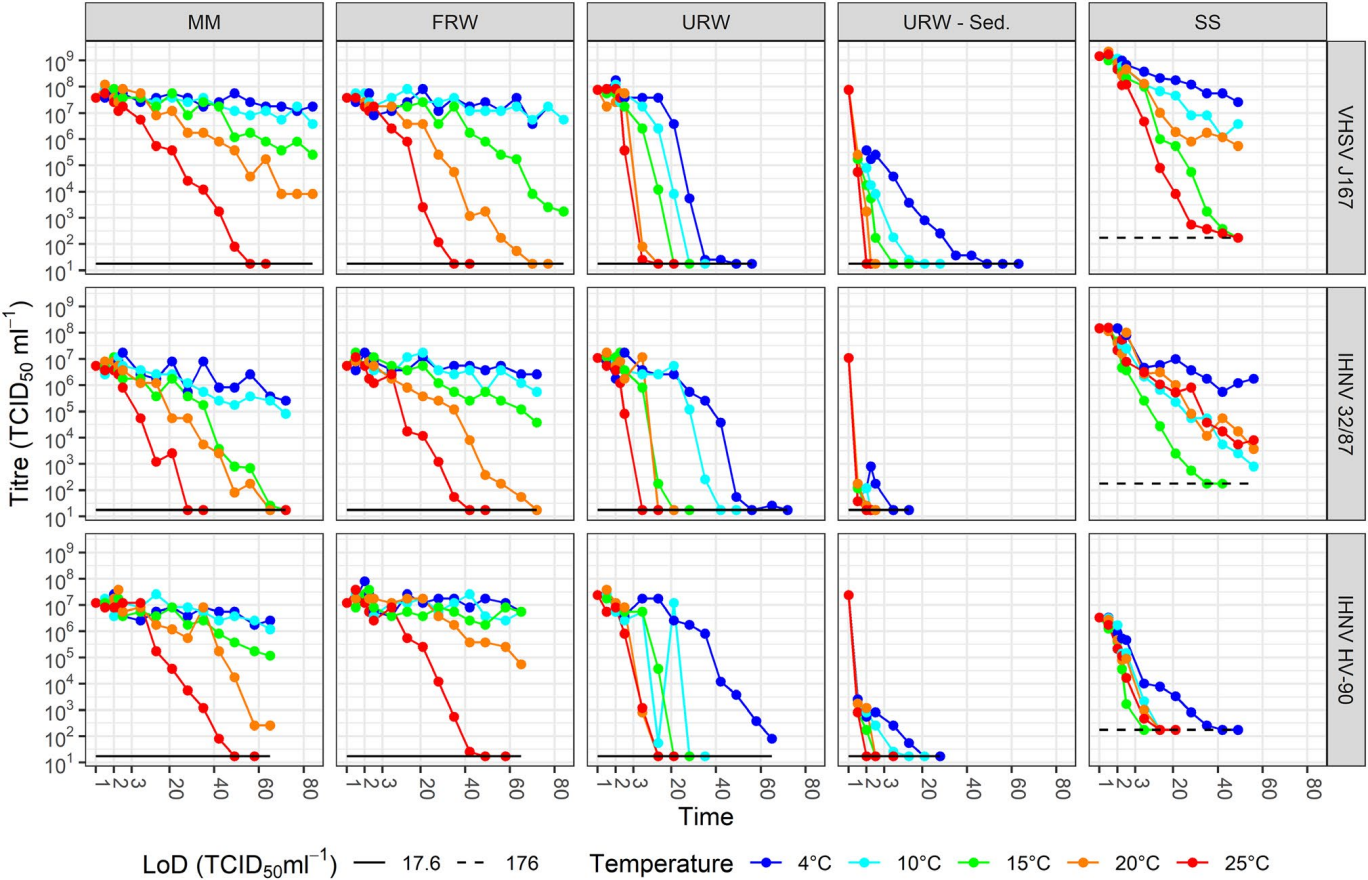
VHSV J167, IHNV 32/87 and IHNV HV‐90 survival (measured as TCID_50_ ml^‐1^) over time in cell culture maintenance medium (MM), filtered river water (FRW), unfiltered river water (URW), water separated from the unfiltered river water‐sediment mixture (URW‐Sed.) and resuspended virus after drying on stainless steel (SS), at 4, 10, 15, 20 and 25°C. The limit of detection (LoD) of the assay is displayed

The survival of VHSV and IHNV over time in cell culture medium, filtered and unfiltered river water, associated with sediment and dried on stainless steel at various incubation temperatures is represented in Figure 2. The survival over time varied between temperatures, matrices and isolates. For precise comparison, detailed results can be found in Table S1.

### 3.1 Temperature effect

The survival times of both VHSV and IHNV decreased as temperature increased in all matrices, excluding virus dried at 15°C on stainless steel (Figure 2 and Table S1). The longest survival time was seen in MM and FRW when incubated at 4 and 10°C, with either retainment of initial titres or minimal decrease (< 1 to 1.83 log_10_ drop) after the maximum time tested (65 to 84 days). The shortest survival in all matrices, except after drying on stainless steel, was at 25°C; viruses were undetectable at the end of the study (6.92, 5.92 and 4.28 log_10_ drop for VHSV J167, IHNV 32/87 and IHNV HV‐90, respectively). The maximum survival at 25°C was in MM or FRW: 49 to 56 days, 35 to 42 days and 42 to 49 days for VHSV J167, IHNV 32/87 and IHNV HV‐90, respectively.

### 3.2 Desiccation on stainless steel

Survival of all virus isolates was longer when dried on the stainless steel than in the URW and the URW‐sediment mixture (Figure 2 and Table S1). The survival at 4 to 25°C ranged between 42 and > 49 days for VHSV J167, 28 to > 56 days for IHNV 32/87 and 3 to 42 days for IHNV HV‐90. VHSV and IHNV isolates that were dried on stainless steel discs showed longest survival at 4°C. At 25°C, VHSV decreased to undetectable levels (6.92 log_10_ drop) between 42 and 49 days and this was also the case at 15°C. IHNV 32/87 decreased the fasted at 15°C and was no longer detected at day 35 (5.92 log_10_ drop) but at 10, 20 and 25°C there was a similar rate of decrease and virus was still detectable at day 56. IHNV HV‐90 had a similar rate of decrease at temperatures between 10 and 25°C; virus was undetectable at day 7 and 14 (4.28 log_10_ drop).

### 3.3 Survival with inorganic, organic content and live biota: MM, FRW, URW, URW‐sediment and fungal growth

Survival at 4 to 25°C in MM ranged between 49 and > 84 days for VHSV J167, 21 to > 72 days for IHNV 32/87 and 42 to > 65 days for IHNV HV‐90 (Figure 2 and Table S1). Survival in FRW ranged between 28 and > 84 days for VHSV J167, 35 to > 72 days for IHNV 32/87 and 42 to > 65 days for IHNV HV‐90. Survival in URW ranged between 7 and 49 days for VHSV J167, 3 to 72 days for IHNV 32/87 and 7 to > 65 days for IHNV HV‐90. Survival in URW‐sediment ranged between 1 hr and 49 days for VHSV J167, 1 hr to 7 days for IHNV 32/87 and 1 hr to 21 days for IHNV HV‐90.

Survival was lower in URW compared to FRW at all temperatures tested. However, titres of virus in FRW and URW samples were within 1 log_10_ of the calculated starting titres 1 hr after spiking, whereas a drop of between 2.47 and 5.47 log_10_ was seen in the water that was separated from the unsterile URW‐sediment mixture. All viruses were undetectable after 1 day in water that was separated from the URW‐sediment at 25°C for all viruses (further studies on survival, adsorption and desorption are described below). At 4 and 10°C, the survival only dropped below detectible levels between 42 and 49 and 14 to 21 days, respectively, for VHSV J167. The decrease was more rapid for IHNV: 3 to 7 and 1 to 2 days, respectively, for IHNV 32/87 and 14 to 21 and 7 to 14 days, respectively, for IHNV HV‐90.

Fungal growth was first visible at the higher temperatures: in URW fungus was present from day 2 at 20 and 25°C, day 3 at 15 and 10°C and day 14 at 4°C. FRW had some fungal growth visible at day 21 at 25 and 20°C and day 49 at 15°C but no fungus at 10 and 4°C. Fungal growth was observed from day 28 at 15°C in the MM inoculated with IHNV 32/87. The results show the viral titre in the MM drop below detectible levels quicker than FRW in this instance (Figure 2 and Table S1). No fungal growth was observed in the MM inoculated with VHSV J167 and IHNV HV‐90.

### 3.4 Soil and sediment—survival, adsorption and desorption

**Figure 3:**
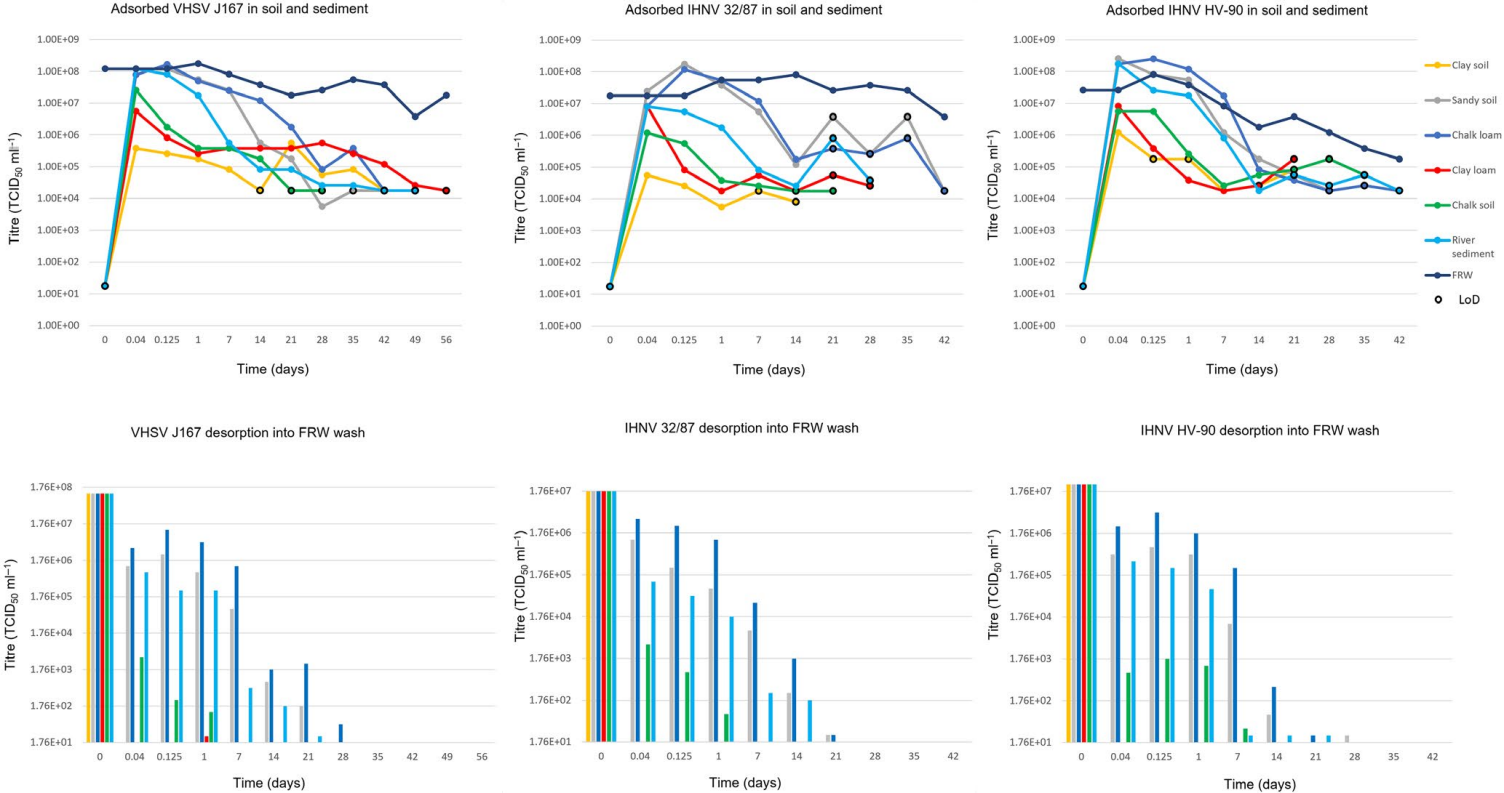
Survival, adsorption and desorption of VHSV J167, IHNV 32/87 and IHNV HV‐90 in soil and sediment. Top charts: survival of adsorbed virus was calculated by ‘titre of FRW‐soil/sediment minus the titre desorbed into the FRW wash’ at each time point. The limit of detection (LoD) varied depending on the soil/sediment restricting cell visibility. Bottom charts: desorption of the viruses was quantified in the FRW wash replaced at each sample point. At time 0, the virus levels before the addition of the soil/sediment were measured. The LoD of the assay was 1.76 × 10^1^ TCID_50_/ml

(Figure 3 and Table S2). Figure 3 shows the number of days the virus adsorbed to the soil and sediment mixtures before it was undetectable and the desorption into the FRW washes (replaced at each sample point). Table S2 shows more detailed data.

### 3.5 Survival and absorption from FRW mixed with sterile soil

After 1 hr, there was an immediate drop in viral titres in the FRW inoculum separated from the five sterile soil and sediment mixtures compared to higher levels in the unseparated soil‐liquid mixture (Figure 3 and Table S2). The highest initial drop in virus titres from the separated FRW, to undetectable levels (≥ 6 logs_10_), was within 1 hr for clay soil and clay loam. After calculating the quantity of adsorbed virus, it was found that virus that adsorbed to clay soil was still infectious but had a 1.33 to 2.5 log_10_ drop in titre after 1 hr compared to that in the spiked FRW wash (initial inoculum) at time 0. After replacing the FRW, there was no desorption of virus into the FRW washes. The FRW‐soil/sediment mixtures of non‐clay‐based soils had a higher titre of adsorbed virus after 1 hr (≤ 1.17 log_10_ drop from the inoculum) even though less virus appeared adsorbed than the clay soils; 8.15x10^2^ (in chalk soil) to 3.78 × 10^6^ (in chalk loam) remained unadsorbed in the FRW inoculum separated after 1 hr.

### 3.6 Survival in soil

Titres slowly decreased in the soil for all isolates, and the VHSV J167 isolate was detected longer than the IHNV isolates in all soils (Figure 3 and Table S2). The longest association of viable virus detected in the soil was VHSV in clay loam; virus was still detected at 49 days. Due to the soil restricting visibility, the maximum log_10_ drop visible was between 2.67 and 4.33 between the viruses from a possible 3.5 to 7.17 log_10_ drop from the absorbed virus. Based on the visible rate of decrease it is likely viable virus remains in all soil types, beyond the point at which it was no longer detectable. Although starting with a higher titre adsorbed to the soil, there were steeper drops in the non‐clay soils and sediment. Based on the rate of decay, it is likely the virus in clay soils will survive the longest, but the detectable limit would have to be lower to confirm this.

### 3.7 Desorption

Apart from VHSV and IHNV adsorbed to clay soil and IHNV adsorbed to clay loam, the other soils and the sediment showed desorption into the weekly washes of FRW (but with decreasing amounts each week) (Figure 3 and Table S2). The longest dissociation observed was the VHSV from chalk loam for 28 to 35 days and sandy soil for 21 to 28 days and IHNV isolates from chalk loam for 21 to 28 days and sandy soil for 28 to 35 days. After time and/or further washes, the virus remained associated and could no longer be washed off.

## 4 DISCUSSION

As environmental conditions vary considerably and there are discrepancies between published data, continued research on the persistence of viruses is necessary for informing disease control risk assessments and emphasise the relevance of strict biosecurity protocols to prevent introductions. Our results assist in one of the evaluating requirements for virus transmission via water published by Oidtmann and colleagues (Oidtmann et al., 2018). We provide additional data on progression of virus decay, which was deemed important in their review on survival of viruses, to aid modelling of potential virus spread via water. The quantification of the virus adsorbed to soils and sediment and desorption over time provides true decay times in soil compared to studies that present survival times in the liquid separated from a water‐soil mixture (underestimating true persistence due to adsorption) and those that assume the decrease in titre in the water separated from the soil is the full adsorption that has taken place. Survival on stainless steel helps highlight the risks and importance of biosecurity that should be in place in many facilities.

Due to the limited number of replicates (a single matrix at each temperature, bar stainless steel which had duplicate steel discs for each time point at each temperature, reducing to one disc after 3 weeks), statistical significances between our findings cannot be provided. This limits the comparisons that can be made between treatments and each virus; however, descriptive data are provided, and qualitative comparisons can be made.

VHSV and IHNV can be very sensitive to ultraviolet light, even in aqueous solutions with high turbidity (Afonso et al., 2012; Ahne, 1982b; Garver et al., 2013; Øye & Rimstad, 2001). Differences can be found between virus types and the suspension it is in (particulate matter can cause a shielding effect which protects the organisms) (Afonso et al., 2012; Jolis et al., 2001; Øye & Rimstad, 2001). The data presented here and, unless otherwise stated, in associated cited literature report viral survival times when incubation was undertaken in the dark in laboratory incubators. As such, they represent the likely upper range of survival times as the virus is protected from the inactivating action of ultraviolet light in natural daylight.

### 4.1 Temperature effect

There was a clear inverse relationship between survival and temperature observed in the river water and sediment matrices chosen to mimic natural environmental conditions. This is consistent with trends observed in previous survival studies across a wide range of viruses including VHSV and IHNV (Hawley & Garver, 2008; Kell et al., 2014; Mori et al., 2002; Parry & Dixon, 1997). Survival at 4°C showed the worst‐case scenario in the temperature range tested, and survival was considerably longer in all matrices compared to 25°C. This temperature has relevance to the colder months of the year and deeper water.

### 4.2 Desiccation on stainless steel

There have been few studies on the survival of VHSV and IHNV after desiccation on surfaces. The survival of VHSV has been studied after drying on plastic (6 to 21 days (or > 15 days) at temperatures between 37°C and 4°C) (Ahne, 1982a; Pham et al., 2011), and survival of ranaviruses has been studied after drying on stainless steel surfaces (estimated persistence of 60 to 80 days for amphibian ranaviruses and 74 to 75 days for reptilian ranaviruses after drying at room temperature) (Nazir et al., 2012), but there are no data available for survival on stainless steel. Stainless steel is a non‐porous material and has a relatively inert smooth surface that facilitates resuspension of dried virus and has high relevance in the aquaculture industry and research facilities, as it is a commonly used for machinery and work surfaces (e.g. grading machines, pumps, fitting and fixtures on holding/transport tanks, hatchery equipment, dip net frames, sorting tables, workbenches, sinks, weighing equipment and laboratory safety cabinets). Until disease is recognized, restrictions will not be enforced and infected pre‐clinical fish may be slaughtered and processed in areas which are free from the pathogen and therefore represent a potential route of introduction on to machinery and work surfaces. (Anon, 2006; Joiner et al., 2020; Oidtmann et al., 2011, 2014).

In this study, survival of both VHSV and IHNV after drying on stainless steel was longer than under the natural environmental conditions mimicked by URW and the URW‐sediment mixtures. The inverse relationship between temperature and survival observed in the other matrices was apparent for VHSV, except for the 15°C samples that decreased at the same rate as the 25°C samples. The IHNV isolates showed very similar survival rates between 10 and 25°C (Figure 2, Table S1). Without repeat experiments, it is not known if this is significant or due to other factors and therefore could be explored further. However, the survival times presented are still beneficial. Given the long survival of both viruses at 4°C on stainless steel, there is clearly potential for contamination on equipment and work surfaces of relevant establishments such as processing facilities, smokeries, fish farms (particularly if they have on site processing facilities), fisheries and indeed in research and diagnostic laboratories, which should be taken into consideration.

### 4.3 Survival in MM, FRW and URW

Cell culture MM was used as a positive control representing most favourable conditions. It provides an indication of the stability of the viruses but unlikely to provide realistic data when relating to survival in the environment. Survival in freshwater was investigated for its relevance to the UK and because it is the most common environment of rainbow trout and the majority of other species susceptible to VHSV and IHNV. Survival in FRW was included to give a ‘worst‐case’ scenario in water, that is how long the virus may survive in clear water under ideal conditions, compared to a more relevant survival time in URW containing organic and inorganic content and live biota.

This study demonstrated that virus survival in FRW was longer than previously published data by de Kinkelin & Scherrer (≤ 1 log_10_ drop for all viruses in 35 to ≥ 65 days at 15°C compared to 1 log_10_ drop within 24 hr at 14°C (de Kinkelin & Scherrer, 1970). In URW, at 15°C the titres of all three virus isolates tested showed similar results to Hawley & Garver for VHSV (this study found a 3.8 log_10_ drop after 14 days at 15°C compared to a 3 log_10_ drop after 13 days at 15°C) (Hawley & Garver, 2008), whereas Kell and co‐workers observed a faster decline for IHNV (4 to 5 days at 15°C) (Kell et al., 2014), and Kamei and co‐workers observed IHNV titre decreased faster still (< 3 days at 15°C) (Kamei et al., 1988a) (Figure 2 and Table S1). At 10°C, the survival in URW (21to 42 days) in this study was slightly less than expected when compared to previous published studies that started with lower titres of virus but persisted longer (49 days) (Ahne, 1982a, 1982b; Wedemeyer et al., 1978).

‘Natural water’ will vary within a country or region, in different parts of a single river system and on a day to day basis. The type of freshwater is of importance in the survival of the viruses (Kamei et al., 1988a; LaPatra et al., 2001; Pietsch et al., 1977; Wedemeyer et al., 1978; Yoshimizu et al., 1986); however, survival of the viruses in ‘natural waters’ is more relevant for risk assessments and disease outbreak modelling than survival data in artificial media.

### 4.4 Survival with inorganic and organic content

As inorganic and organic content in the environmental test matrices increased, virus survival decreased. URW had more organic and inorganic content than FRW and URW‐sediment had more than URW.

A complication in using ‘natural waters’ and soils is that they may contain factors that inactivate the viruses. The effect of micro‐organisms has been widely published, and our results are in accordance with many of these, where survival decreases in non‐sterile matrices compared to sterile matrices (Hawley & Garver, 2008; Kamei et al., 1988b; LaPatra et al., 2001; Mori et al., 2002; Nazir et al., 2010; Oidtmann et al., 2018; Sobsey et al., 1980; Yoshimizu et al., 1986). However, synergistic interactions between viruses and bacteria have also been reported: in some cases bacteria and proteinaceous materials present in waste water have been shown to stabilise viruses, protecting them from degredation or acting as a vector, facilitating infection or even promoting genetic recombination of the virus, but in others no effect has been observed (Yates et al., 2000; Jones et al., 2014; Uchiyama et al., 2014; Wilks et al., 2015; Erickson et al., 2018). The variance between our data and others is hard to quantify unless extensive and expensive replicative longitudinal studies are undertaken. Fungus contamination is likely to have influenced the survival of samples of IHNV 32/87 in MM in this study, and the longest survival may have occurred in MM for this isolate, rather than FRW, had this contamination not occurred.

Several mechanisms by which micro‐organisms may influence virus inactivation have been suggested and reviewed; these include proteolytic enzymes, photosensitizers and oxidizing or reducing agents (Yates et al., 2000). Organic matter (both soil‐associated and dissolved) is known to weaken the electrostatic binding of viruses to soils (Kimura et al., 2008). It has been suggested that micro‐organisms may enhance virus inactivation in two ways—one is by interfering with virus adsorption to soil particles, the other is by the production of a soluble metabolic product that is virucidal (Lipson & Stotzky, 1985). These factors may account for some of the discrepancies seen between our results and published data previously mentioned. The river Wey at the point of our sampling is a spring‐fed chalk stream, only two kilometres from source and as such is relatively clear and clean.

### 4.5 Survival, adsorption and desorption in URW‐sediment and FRW‐soil and sediment mixtures

Any association with soil and sediment may play a major role in viral hydrotransportation and survival (LaBelle & Gerba, 1979, 1980) and in protecting them against thermoinactivation (Liew & Gerber, 1980) but conducting comparable survival studies in soils and sediment can be difficult. There must be a separation of the effect of the sediment from the amount of moisture it contains and associated micro‐organisms (discussed above), as well as the effect of adsorption of the viruses to the constituents of the sediment which would reduce the apparent titre of the virus and hence over‐estimate the loss of titre of the virus.

The first assessments of survival in this study were in URW‐sediment (with no removal of the liquid after adsorption) at temperatures ranging between 4 and 25°C. Viral titres decreased quickest at all temperatures in the water sample separated from the URW‐sediment. The sharp drops in virus titres in the separated liquid, 0 to 1 hr after addition of the virus to sediment at all temperatures tested (Figure 2 and Table S1), suggested initial adsorption or rapid inactivation from components in the sediment; therefore, survival, adsorption and desorption were investigated further in sterile soils and sediment at 4°C to confirm this.

There are no studies to compare VHSV inactivation in different sediments. One study has been published providing data on the survival of VHSV in a cell culture medium‐unsterile pond sludge mixture by quantifying the virus in the separated medium (Ahne, 1982b), and two studies have been published providing data on the survival of IHNV in the water portion of an unsterile marine sediment mixture and sediment from a freshwater fish farm (Kamei et al., 1987; Kamei et al., 1988a), but it is unknown if the quantities previously published are due to survival or adsorption. There is only one publication that investigated IHNV adsorbed to the soil portion (Yoshinaka et al., 2000) and is discussed below.

In this study, soil and sediment samples used to assess ‘survival, adsorption and desorption’ were autoclaved to avoid possible effects of interfering live biota mentioned above but the components of each soil were not measured. Soils contain a mixture of minerals and organic matter, and organic soils tend not to bind as much virus as mineral soils (Rao & Melnick, 1986). Normal (mineral) soils contain between 1% and 6% organic matter (https://www.iscc‐system.org/wp‐content/uploads/2018/09/Clay‐sandy‐and‐peat‐soils‐and‐soil‐organic‐matter.pdf accessed 28 August 2020). Incubation was at 4°C to give a worst‐case scenario within the range of temperatures already studied. An elution process such as that published for enteric human viruses (Gerba et al., 1977) was not used as a high and low pH affects the titre of the viruses (Dixon et al., 2012).

The viruses fully decreased in the FRW inoculum separated from the clay soil and clay loam after 1 hr yet were detectable in the FRW‐clay soil and FRW‐clay loam mixtures. When calculating how much was adsorbed to the clays, the titre in the separated FRW inoculum was taken away from the titre in the FRW‐clay soil/loam. These gave a lower titre than the initial inoculum so it can be assumed that within the first hour there may have been some inactivation. As the FRW inoculum was then removed and there was no desorption of virus into the further washes, the titres are an accurate representation of what remains adsorbed in these soils over time.

The non‐clay‐based samples had higher levels of virus left in the FRW inoculum after 1 hr of mixing, but higher levels of adsorbed virus calculated in the soils and sediment than the clay‐based soils, so the initial inactivation seen in the clay‐based soils is not evident. The soils adsorbed virus with ≤ 1.17 log_10_ drop from the initial inoculum. The viruses desorbed into the washes and as such caused a faster decline in the adsorbed virus compared to that adsorbed to the clay‐based soils (that did not desorb further). The ‘adsorbed’ virus presented in these soils is therefore decreasing due to a combination of desorption and inactivation. The differing quantities desorbed imply the soil type is a determining factor in viral desorption. It should also be remembered that some desorbed virus may also have been inactivated during the weekly sample points.

Clay soils have very small particles (< 0.002 mm) and because of this, initial adsorption may have been less as the water stays on top of the soil and did not move inside as easily. Desorption is less likely because the water moves through very slowly, so the clay soils have a very good water holding capacity (the particles are so small, the water is trapped between them). Sandy soils have relatively large particles (0.05 to 0.2 mm) so could explain the opposite effect, high starting adsorption with a long desorption and rapid decrease in adsorbed virus. Loamy soils have a mixture of particles so would sit somewhere in the middle—this is less clear with the results but may have become more apparent over time (https://www.iscc‐system.org/wp‐content/uploads/2018/09/Clay‐sandy‐and‐peat‐soils‐and‐soil‐organic‐matter.pdf accessed 27 August 2020). Chattopadhyay and Puls suggested that viruses adsorb to colloidal clays using van der Waals forces. The pH influences the charge on both viruses and soils, and ionic strength and its constituents determine the binding forces between them (Chattopadhyay & Puls, 2000). Other factors include concentration of ions, cation‐exchange capacity (water hardness, i.e. Ca2 + and Mg2+), type and amount of clay, organic matter concentration, proteins, isoelectric point (IEP) and virus type and strain (Fillhart et al., 1998; Kimura et al., 2008). These variables may explain some differences observed with each soil type.

VHSV was detectible for the longest time adsorbed to clay loam, even after an initial drop in adsorbed titre, but varying limits of detection (soil obstructing the view of the cells) prevents us knowing how long survival was past the last detection. Our detection of all the adsorbed viruses is likely to be an underestimate of survival based on the rate of decay; however, the rate of decay is useful for disease models. Based on the rate of decay (the log_10_ decrease of adsorbed virus), virus in the clay soils is likely to have survived the longest, and there appears to be differences in the decay depending on soil type that may have been more or less pronounced over time. Survival and adsorption may also differ between viruses; this also may have been more evident over time. Previous studies on the adsorption of different viruses to soils have shown a high degree of variability not only among different virus types but among different strains of the same virus type (Kimura et al., 2008; Yates et al., 2000).

There has been one study that also investigated the survival of IHNV in tris‐HCl‐soil mixtures by observing presence or absence of IHNV in both the supernatant and the complete mixture (Yoshinaka et al., 2000). IHNV was still detected 9 weeks (63 days) after adsorption to kaolin (clay), Japanese acid clay and diatomaceous earth compared to our results detecting VHSV J167 at day 35 and day 49, IHNV 32/87 at day 1 and day 14 and IHNV HV‐90 at day 7 and day 7 in clay soil and clay loam, respectively. This study only detected a 0.67 to 2.67 log_10_ decrease in adsorbed virus, suggesting survival may have continued to beyond 63 days as with the published study. Yoshinaka and co‐workers did not remove the initial inoculum so there may have been continual adsorption and desorption within their sample mixtures. By removing the inoculum after 1‐hr adsorption and replacing the water to measure desorption, the results provide a more realistic representation of the environment, as water flow and virus desorption can play a role in virus transmission. The published study also demonstrated that an IHNV suspension mixed with diatomaceous earth and different types of clay could cause mortality in rainbow trout fry (Yoshinaka et al., 2000). As we have demonstrated desorption does not occur after 1 hr in clay soil, it is assumed their infection was caused by the adsorbed virus, although the time between mixing the virus with the soil to adding it to the fish is not published.

Due to the apparent adsorption, percolation in soil (particularly clay‐based soil) may appear to be an appropriate method for waste water sanitation from processing facilities, as concluded by Skall and co‐workers for VHSV (Skall et al., 2015). However, within the confines of this study we are not able to comment on the relative influence of adsorption and or inactivation of virus on the retention they observed or the likely impact of higher viral loading. The survival of adsorbed virus and desorption depending on soil type would be important if considering this method. This study shows that there are associated risks with soils, as the virus can remain infectious for several weeks. In the context of an outbreak situation, the virus could remain present in earth ponds or downstream of infected fish farms for weeks after infected fish have been removed, even when it is no longer detectible in the surrounding water. In addition, there is also the risk that even after adsorption, the adsorbed virus is not necessarily permanently immobilized. Desorption can also continue in some soil types for several weeks, and a change in ionic strength or salt concentration of the surrounding medium, such as would be induced by a rainfall event, could cause further virus desorption, allowing further transport in the soil (Yates et al., 2000). These factors and associated risks must be considered when modelling disease outbreak situations or considering biosecurity measures. The fallow period for farms infected with IHNV and VHSV should be at least at least 6 weeks after the farm was emptied, cleansed and disinfected according to the EU diagnostic manual (EU Commission, 2015). The differences in survival times between viruses and soil types can help inform these control measures.

### 4.6 Conclusions

Survival ranged between 1 hr and > 84 days under different conditions: in MM VHSV survived 49 to > 84 days and IHNV 21 to > 72 days; in FRW VHSV survived 28 to > 84 days and IHNV 35 to > 72 days; in URW VHSV survived 7 to 49 days and IHNV 3 to > 65 days (or 72 days); in the water separated from URW‐Sediment mixtures VHSV survived 1 hr to 49 days and IHNV 1 hr to 21 days, when dried on stainless steel VHSV survived 42 to > 49 days and IHNV 3 to > 56 days, at temperatures ranging from 25 to 4°C. Within the tested range, the lower half of the temperature range has the longest survival and is particularly relevant when considering water temperature in relation to UK climate and deeper water. This study has demonstrated that a range of environmental parameters influence the survival of viruses and machinery and surfaces made of stainless steel should be cleaned and disinfected to prevent potential spread of disease. Persistence of VHSV J167, IHNV 32/87 and IHNV HV‐90 was strongly dependent on temperature, presence of organic and inorganic content, live biota and type of soil association, and although not tested here, it is known that UV is a strong inactivating factor even in turbid water (Afonso et al., 2012; Ahne, 1982b; Garver et al., 2013; Øye & Rimstad, 2001). In real aquatic systems, this is likely to translate into longer persistence of virus in clear streams or rivers with gravel beds and plenty of shade compared to muddy lake water or fish rearing water with high amounts of suspended solids (e.g. fish faeces and feed remains) particularly after a storm. In the context of an IHNV or VHSV disease outbreak, removal of live fish is one of the obvious first steps to prevent further virus from being released into the environment. However, our study clearly demonstrates that virus would be likely to remain in the environment for weeks or months. Survival of the viruses in the environment outside the optimum temperatures for disease in rainbow trout show how virus can persist and implies disease may reoccur as seasonal changes take place and temperatures change. The information provided here will inform measures required to contain IHNV or VHSV outbreaks. Data on survival of these viruses in the environment can be used to plan necessary steps to decontaminate infected sites (e.g. through fallowing, disinfection and sediment removal) and assess risk of further disease spread (downstream (via water) or via fomites). ‘Quantitative estimates of relevance’ of waterborne and mechanical transmission have previously been generated through expert consultation by grading risks according to importance (Oidtmann et al., 2014). This study presents experimental data to inform ‘quantitative estimates for pathogen spread’ via these routes and will inform import risk assessments, support planning of control measures and inform appropriate biosecurity measures.

### 4.7 Further research

Other information required for assessing virus transmission by water includes (a) virus strain; (b) viral shedding rates per animal or biomass per time unit; (c) prevalence of infection in the shedding aquatic animal population over the course of a disease outbreak; (d) water flow rates, which will dilute virus concentrations; and (e) minimum infectious dose for exposed naive fish (Oidtmann et al., 2018). Further research will be required to fill some of these data gaps.

## CONFLICT OF INTEREST

The authors confirm that they have no conflict of interest to declare.

## ETHICAL STATEMENT

The authors confirm that the ethical policies of the journal, as noted on the journal's author guidelines page, have been adhered to. No ethical approval was required as this study was in vitro and no animals were used in this study.

## Supporting information

Table S1

Table S2

## Data Availability

The data that support the findings of this study are available in the supplementary material of this article.

## References

[tbed13888-bib-0001] Afonso, L. O. B., Richmond, Z., Eaves, A. A., Richard, J., Hawley, L., & Garver, K. A. (2012). Use of ultraviolet C (UVC) radiation to inactivate infectious hematopoietic necrosis virus (IHNV) and viral haemorrhagic septicaemia virus (VHSV) in fish processing plant effluent. Journal of Aquaculture Research & Development, 3(1), 1–5. 10.4172/2155-9546.1000120

[tbed13888-bib-0002] Ahne, W. (1982a). Untersuchungen zur Tenazität der Fischviren. Fortschr Veterinärmed, 35, 305–309.

[tbed13888-bib-0003] Ahne, W. (1982b). Vergleichende Untersuchungen über die Stabilität von vier fischpathogen Viren (VHSV, PFR, SVCV, IPNV). Zentralbl Veterinarmed B, 29, 457–476. 7148214

[tbed13888-bib-0004] Anon (1991). Council Directive 91/67/EEC (repealed) of 28 January 1991 concerning the animal health conditions governing the placing on the market of aquaculture animals and products. Official Journal of the European Union. Directives originating from the EU 1991 No. 67. Retrieved from http://www.legislation.gov.uk/eudr/1991/67/contents (10 May 2020).

[tbed13888-bib-0005] Anon (2006). Council Directive 2006/88/EC of 24 October 2006 on animal health requirements for aquaculture animals and products thereof, and on the prevention and control of certain diseases in aquatic animals. Official Journal of the European Union, L328, 14.

[tbed13888-bib-0006] Anon (2009). 2009/177/EC: Commission decision of 31 October 2008 implementing Council Directive 2006/88/EC as regards surveillance and eradication programmes and disease‐free status of Member States, zones and compartments *(notified under document number C (2008) 6264*) (Text with EEA relevance). Official Journal of the European Union, L63, 15.

[tbed13888-bib-0007] Asl, A. H. K., Soltani, M., Kazemi, B., Haghdoust, S., & Sharifpour, I. (2007). Use of Immunohistochemical and PCR Methods in Diagnosis of Infectious Haematopoietic Necrosis Disease in some Rainbow Trout Hatcheries in Iran. Pakistan Journal of Biological Sciences, 10(2), 230–234. 10.3923/pjbs.2007.230.234 19070020

[tbed13888-bib-0008] Bootland, L. M., & Leong, J. C. (1999). Infectious hematopoietic necrosis virus. In P. T. K. Woo, & D. W. Bruno (Eds.), Fish Diseases and Disorders, Volume 3: Viral, Bacterial and Fungal Infections (pp. 57–121). CAB International.

[tbed13888-bib-0009] Chattopadhyay, S., & Puls, R. W. (2000). Forces dictating colloidal interactions between viruses and soil. Chemosphere, 41(8), 1279–1286. 10.1016/s0045-6535(99)00519-6 10901259

[tbed13888-bib-0010] Cieslak, M., Wahli, T., Diserens, N., Haenen, O. L. M., & Schütze, H. (2017). Phylogeny of the Infectious Hematopoietic Necrosis Virus in European Aquaculture. PLoS One, 12(9), e0184490. 10.1371/journal.pone.0184490 28886189 PMC5590938

[tbed13888-bib-0011] de Kinkelin, P., Hattenberger, A. M., Torchy, C., & Lieffrig, F. (1987). Infectious haematopoietic necrosis (IHN): First report in Europe. European Association of Fish Pathologists. In Third International Conference (Abstract 57).

[tbed13888-bib-0012] De Kinkelin, P., & Scherrer, R. (1970). Le virus d’Egtved. I. Stabilité, développement et structure du virus de la souche enoise F1. Annales De Recherches Veterinaires, 1(1), 17–30.

[tbed13888-bib-0013] Dixon, P., Paley, R., Alegria‐Moran, R., & Oidtmann, B. (2016). Epidemiological characteristics of Infectious hematopoietic necrosis virus (IHNV) – a review. Veterinary Research, 47(1), 63. 10.1186/s13567-016-0341-1 27287024 PMC4902920

[tbed13888-bib-0014] Dixon, P. F., Smail, D. A., Algoet, M., Hastings, T., Bayley, A., Byrne, H., Dodge, M., Garden, A., Joiner, C., Roberts, E., Verner‐Jeffrys, D., & Thompson, F. (2012). Studies on the effect of temperature and pH on the inactivation of fish viral and bacterial pathogens. Journal of Fish Diseases, 35(1), 51–64. 10.1111/j.1365-2761.2011.01324.x 22168455

[tbed13888-bib-0015] Einer‐Jensen, K., Ahrens, P., Forsberg, R., & Lorenzen, N. (2004). Evolution of the fish rhabdovirus viral haemorrhagic septicaemia virus. Journal of General Virology, 85(5), 1167–1179. 10.1099/vir.0.79820-0 15105533

[tbed13888-bib-0016] Enzmann, P. J., Castric, J., Bovo, G., Thiery, R., Fichtner, D., Schütze, H., & Wahli, T. (2010). Evolution of Infectious Hematopoietic Necrosis Virus (IHNV), a Fish Rhabdovirus, in Europe over 20 Years: Implications for Control. Diseases of Aquatic Organisms, 89(1), 9–15. 10.3354/dao02182 20391908

[tbed13888-bib-0017] EU Commission (2015). Commission Implementing Decision (EU) 2015/1554 of 11 September 2015 laying down rules for the application of Directive 2006/88/EC as regards requirements for surveillance and diagnostic methods (ed Union E). Official Journal of the European Union, L247, 241–262.

[tbed13888-bib-0018] Fijan, N., Sulimanovic, D., Bearzotti, M., Muzinic, D., Zwillenberg, L. O., Chilmonczyk, S., Vautherot, J. F., & de Kinkelin, P. (1983). Some properties of the *Epithelioma papulosum cyprini* (EPC) cell line from carp *Cyprinus carpio* . Annales de l'Institut Pasteur / Virologie, 134(2), 207–220. 10.1016/S0769-2617(83)80060-4

[tbed13888-bib-0019] Fillhart, R. C., Bachand, G. D., & Castello, J. D. (1998). Detection of infectious tobamoviruses in forest soils. Applied and Environment Microbiology, 64(4), 1430–1435. 10.1128/AEM.64.4.1430-1435.1998 PMC106165 16349545

[tbed13888-bib-0020] Garver, K. A., Mahony, A. A. M., Stucchi, D., Richard, J., Van Woensel, C., & Foreman, M. (2013). Estimation of parameters influencing waterborne transmission of infectious hematopoietic necrosis virus (IHNV) in Atlantic salmon (*Salmo salar*). PLoS One, 8(12), e82296. 10.1371/journal.pone.0082296 24340016 PMC3855332

[tbed13888-bib-0021] Gerba, C. P., Smith, E. M., & Melnick, J. L. (1977). Development of a quantitative method for detecting enteroviruses in estuarine sediments. Applied and Environment Microbiology, 34(2), 158–163. 10.1128/aem.34.2.158-163.1977 PMC242615 20839

[tbed13888-bib-0023] Hawley, L. M., & Garver, K. A. (2008). Stability of viral hemorrhagic septicemia virus (VHSV) in freshwater and seawater at various temperatures. Diseases of Aquatic Organisms, 82, 171–178. 10.3354/dao01998 19244968

[tbed13888-bib-0024] He, M., Ding, N. Z., He, C. Q., Yan, X. C., & Teng, C. B. (2013). Dating the divergence of the infectious hematopoietic necrosis virus. Infection, Genetics and Evolution, 18, 145–150. 10.1016/j.meegid.2013.05.014 23722020

[tbed13888-bib-0025] Joiner, C., Teixeira, A. M., & Oidtmann, B. (2020). Shedding of viral haemorrhagic septicaemia virus (VHSV) from rainbow trout, Oncorhynchus mykiss, peaks prior to onset of clinical signs and can be quantified in liquid and solid waste from processing pre‐clinical fish. Manuscript in progress.

[tbed13888-bib-0026] Jolis, D., Lam, C., & Pitt, P. (2001). Particle effects on ultraviolet disinfection of coliform bacteria in recycled water. Water Environment Research, 73(2), 233–236. 10.2175/106143001X139218 11563383

[tbed13888-bib-0027] Kamei, Y., Yoshimizu, M., Ezura, Y., & Kimura, T. (1987). Effects of estuarine and marine waters on the infectivities of infectious hematopoietic necrosis virus (IHNV) and infectious pancreatic necrosis virus (IPNV). Bulletin of the Faculty of Fisheries, Hokkaido University, 38, 271–285.

[tbed13888-bib-0028] Kamei, Y., Yoshimizu, M., Ezura, Y., & Kimura, T. (1988a). Effects of environmental water on the infectivities of infectious hematopoietic necrosis virus (IHNV) and infectious pancreatic necrosis virus (IPNV). Journal of Applied Ichthyology, 4(1), 37–47. 10.1111/j.1439-0426.1988.tb00546.x

[tbed13888-bib-0029] Kamei, Y., Yoshimizu, M., Ezura, Y., & Kimura, T. (1988b). Screening of bacteria with antiviral activity from fresh water salmonid hatcheries. Microbiology and Immunology, 32(1), 67–73. 10.1111/j.1348-0421.1988.tb01366.x 3374405

[tbed13888-bib-0030] Kärber, G. (1931). Beitrag zur kollektiven Behandlung pharmakologischer Reihenversuche. Archiv für experimentelle Pathologie und Pharmakologie, 162, 480–483. 10.1007/BF01863914.

[tbed13888-bib-0031] Kell, A. M., Wargo, A. R., & Kurath, G. (2014). Viral fitness does not correlate with three genotype displacement events involving infectious hematopoietic necrosis virus. Virology, 464, 146–155. 10.1016/j.virol.2014.07.003 25068402 PMC4157104

[tbed13888-bib-0032] Kimura, M., Jia, Z.‐J., Nakayama, N., & Asakawa, S. (2008). Ecology of viruses in soils: Past, present and future perspectives. The Journal of Soil Science and Plant Nutrition, 54, 1–32. 10.1111/j.1747-0765.2007.00197.x

[tbed13888-bib-0033] LaBelle, R. L., & Gerba, C. P. (1979). Influence of pH, salinity, and organic matter on the adsorption of enteric viruses to estuarine sediment. Applied and Environment Microbiology, 38(1), 93–101. 10.1128/aem.38.1.93-101.1979 PMC243441 39508

[tbed13888-bib-0034] LaBelle, R. L., & Gerba, C. P. (1980). Influence of estuarine sediment on virus survival under field conditions. Applied and Environment Microbiology, 39(4), 749–755. 10.1128/aem.39.4.749-755.1980 PMC291414 6246838

[tbed13888-bib-0035] LaPatra, S. E., Lauda, K. A., & Morton, A. W. (1991). Antigenic and virulence comparison of eight isolates of infectious hematopoietic necrosis virus from the Hagerman Valley, Idaho, USA. In: Proceedings of the Second International Symposium on Viruses of Lower Vertebrates. Oregon State University Press, Corvallis, p. 125‐132.

[tbed13888-bib-0036] LaPatra, S., Troyer, R. M., Shewmaker, W., Jones, G., & Kurath, G. (2001). Understanding aquatic animal virus survival and trafficking and its role in risk assessment. In: Proceedings of an International Conference: Risk analysis in aquatic animal health. World Organisation for Animal Health, Paris 251‐258.

[tbed13888-bib-0037] Liew, P. F., & Gerber, C. P. (1980). Thermostabilization of enteroviruses by estuarine sediment. Applied and Environment Microbiology, 40(2), 305–308. 10.1128/aem.40.2.305-308.1980 PMC291571 6258475

[tbed13888-bib-0038] Lipson, S. M., & Stotzky, G. (1985). Specificity of virus adsorption to clay minerals. Canadian Journal of Microbiology, 31(1), 50–53. 10.1139/m85-011 3986713

[tbed13888-bib-0039] Mori, K., Iida, H., Nishizawa, T., Arimoto, M., Nakajima, K., & Muroga, K. (2002). Properties of viral hemorrhagic septicemia virus (VHSV) isolated from Japanese flounder *Paralichthys olivaceus* . Fish Pathology, 37(4), 169–174. 10.3147/jsfp.37.169

[tbed13888-bib-0040] Nazir, J., Haumacher, R., Abbas, M. D., & Marschang, R. E. (2010). Use of filter carrier technique to measure the persistence of avian influenza viruses in wet environmental conditions. Journal of Virological Methods, 170(1–2), 99–105. 10.1016/j.jviromet.2010.09.007 20833205

[tbed13888-bib-0041] Nazir, J., Spengler, M., & Marschang, R. E. (2012). Environmental persistence of amphibian and reptilian ranaviruses. Diseases of Aquatic Organisms, 98(3), 177–184. 10.3354/dao02443 22535867

[tbed13888-bib-0042] Oidtmann, B., Dixon, P., Way, K., Joiner, C., & Bayley, A. (2018). Risk of waterborne spread– review of survival of relevant fish and crustacean viruses in the aquatic environment and implications for control measures. Reviews in aquaculture, 10(3), 641–669. 10.1111/raq.12192

[tbed13888-bib-0043] Oidtmann, B., Joiner, C., Stone, D., Dodge, M., Reese, R. A., & Dixon, P. (2011). Viral load of various tissues of rainbow trout challenged with viral haemorrhagic septicaemia virus at various stages of disease. Diseases of Aquatic Organisms, 93, 93–104. 10.3354/dao02298 21381515

[tbed13888-bib-0044] Oidtmann, B. C., Peeler, E. J., Thrush, M. A., Cameron, A. R., Reese, R. A., Pearce, F. M., Dunn, P., Lyngstad, T. M., Tavornpanich, S., Brun, E., & Stärk, K. D. C. (2014). Expert consultation on risk factors for introduction of infectious pathogens into fish farms. Preventative veterinary medicine, 115(3–4), 238–254. 10.1016/j.prevetmed.2014.03.017 24780587

[tbed13888-bib-0045] Øye, A. K., & Rimstad, E. (2001). Inactivation of infectious salmon anaemia virus, viral haemorrhagic septicaemia virus and infectious pancreatic necrosis virus in water using UVC irradiation. Diseases of Aquatic Organisms, 48, 1–5. 10.3354/dao048001 11843135

[tbed13888-bib-0046] Parry, L., & Dixon, P. F. (1997). Stability of nine viral haemorrhagic septicaemia virus (VHSV) isolates in seawater. Bulletin of the European Association of Fish Pathologists, 17, 31–36.

[tbed13888-bib-0047] Pham, P. H., Jung, J., & Bols, N. C. (2011). Using 96‐well tissue culture polystyrene plates and a fluorescence plate reader as tools to study the survival and inactivation of viruses on surfaces. Cytotechnology, 63(4), 385–397. 10.1007/s10616-011-9355-8 21512821 PMC3140832

[tbed13888-bib-0048] Pietsch, J. P., Amend, D. F., & Miller, C. M. (1977). Survival of infectious hematopoietic necrosis virus held under various environmental conditions. Journal of the Fisheries Research Board of Canada, 34(9), 1360–1364. 10.1139/f77-195

[tbed13888-bib-0049] Rao, V. C., & Melnick, J. L. (1986). Environmental virology. American Society for Microbiology.

[tbed13888-bib-0050] Skall, H. F., Jørgensen, C., & Olesen, N. J. (2015). Evaluation of the effect of percolation and NaCl solutions on viral haemorrhagic septicaemia virus (VHSV) under experimental conditions. Aquaculture, 448, 507–511. 10.1016/j.aquaculture.2015.06.032

[tbed13888-bib-0051] Snow, M., Bain, N., Black, J., Taupin, V., Cunningham, C. O., King, J. A., Skall, H. F., & Raynard, R. S. (2004). Genetic population structure of marine viral haemorrhagic septicaemia virus (VHSV). Dis Aquat Org, 61(1–2), 11–21. 10.3354/dao061011 15584406

[tbed13888-bib-0052] Sobsey, M. D., Dean, C. H., Knuckler, M. E., & Wagner, R. A. (1980). Interactions and survival of enteric viruses in soil materials. J Appl Environ Microbiol, 40(1), 92–101. 10.1128/AEM.40.1.92-101.1980 PMC291530 6250478

[tbed13888-bib-0053] Stone, D. M., Ferguson, H. W., Tyson, P. A., Savage, J., Wood, G., Dodge, M. J., Woolford, G., Dixon, P. F., Feist, S. W., & Way, K. (2008). The first report of viral haemorrhagic septicaemia in farmed rainbow trout, *Oncorhynchus mykiss* (Walbaum), in the United Kingdom. Journal of Fish Diseases, 31(10), 775–784. 10.1111/j.1365-2761.2008.00951.x 18681899

[tbed13888-bib-0055] Wedemeyer, G. A., Nelson, N. C., & Smith, C. A. (1978). Survival of the salmonid viruses infectious hematopoietic necrosis (IHNV) and infectious pancreatic necrosis (IPNV) in ozonated, chlorinated, and untreated waters. Journal of the Fisheries Research Board of Canada, 35(6), 875–879. 10.1139/f78-140

[tbed13888-bib-0056] Winton, J., Batts, W., deKinkelin, P., LeBerre, M., Bremont, M., & Fijan, N. (2010). Current lineages of the epithelioma papulosum cyprini (EPC) cell line are contaminated with fathead minnow, *Pimephales promelas*, cells. Journal of Fish Diseases, 33(8), 701–704. 10.1111/j.1365-2761.2010.01165.x 20497291

[tbed13888-bib-0057] World Organisation for Animal Health OIE (2019) Aquatic Animal Health Code 2019, Chapter 2.3.10, Viral haemorrhagic septicaemia; Chapter 2.3.4, Infectious hematopoietic necrosis, Manual of Diagnostic Tests for Aquatic animals, OIE, Paris. Retrieved from http://www.oie.int/international‐standard‐setting/aquatic‐manual/access‐online/ (17 February 2020).

[tbed13888-bib-0058] Yates, M. V., Jury, W. A., Yates, S. R., Anderson, D. L., Stark, L. M., & Sherblom, P. (2000). Measurement of virus and indicator survival and transport in the subsurface (pp. 41–52). AWWA Research foundation and American Water Works Association.

[tbed13888-bib-0059] Yoshimizu, M., Takizawa, H., Kamei, Y., & Kimura, T. (1986). Interaction between fish pathogenic viruses and microorganisms in fish rearing water: Survival and inactivation of infectious pancreatic necrosis virus, infectious hematopoietic necrosis virus an *Oncorhynchus masou* virus in rearing water. Fish Pathology, 21(4), 223–231. 10.3147/jsfp.21.223

[tbed13888-bib-0060] Yoshinaka, T., Yoshimizu, M., & Ezura, Y. (2000). Adsorption and infectivity of infectious hematopoietic necrosis virus (IHNV) with various solids. Journal of Aquatic Animal Health, 12(1), 64–68. 10.1577/1548-8667(2000)012<0064:AAIOIH>2.0.CO;2 28880780

[tbed13888-bib-0062] Erickson, A. K., Mayer, M. J., Narbad, A., Winter, S. E., & Pfeiffer, J. K. (2018). Bacteria Facilitate Enteric Virus Co‐infection of Mammalian Cells and Promote Genetic Recombination. Cell Host Microbe, 23(1), 77–88.e5. 10.1016/j.chom.2017.11.007. 29290575 PMC5764776

[tbed13888-bib-0063] Jones, M. K., Watanabe, M., Zhu, S., Graves, C. L., Keyes, L. R., Grau, K. R., Gonzalez‐Hernandez, M. B., lovine, N. M., Wobus, C. E., Vinjé, J., Tibbetts, S. A., Wallet, S. M., & Karst, S. M. (2014). Enteric bacteria promote human and mouse norovirus infection of B cells. Science, 346(6210), 755–759. 10.1126/science.1257147. 25378626 PMC4401463

[tbed13888-bib-0061] Uchiyama, R., Chassaing, B., Zhang, B., & Gewirtz, A. T. (2014). Antibiotic Treatment Suppresses Rotavirus Infection and Enhances Specific Humoral Immunity. J Infect Dis, 210(2), 171–182. 10.1093/infdis/jiu037. 24436449 PMC4399425

[tbed13888-bib-0064] Wilks, J., Lien, E., Jacobson, A. N., Fischbach, M. A., Qureshi, N., Chervonsky, A. V., & Golovkina, T. V. (2015). Mammalian Lipopolysaccharide Receptors Incorporated into the Retroviral Envelope Augment Virus Transmission. Cell Host Microbe, 18(4), 456–462. 10.1016/j.chom.2015.09.005. 26468748 PMC4795803

